# Gamma-Tocotrienol Modulates Total-Body Irradiation-Induced Hematopoietic Injury in a Nonhuman Primate Model

**DOI:** 10.3390/ijms232416170

**Published:** 2022-12-18

**Authors:** Tarun K. Garg, Sarita Garg, Isabelle R. Miousse, Stephen Y. Wise, Alana D. Carpenter, Oluseyi O. Fatanmi, Frits van Rhee, Vijay K. Singh, Martin Hauer-Jensen

**Affiliations:** 1 UAMS Myeloma Center, University of Arkansas for Medical Sciences, Little Rock, AR 72205, USA; 2 Division of Radiation Health, Department of Pharmaceutical Sciences, University of Arkansas for Medical Sciences, Little Rock, AR 72205, USA; 3 Department of Biochemistry and Molecular Biology, University of Arkansas for Medical Sciences, Little Rock, AR 72205, USA; 4 Division of Radioprotectants, Department of Pharmacology and Molecular Therapeutics, F. Edward Hébert School of Medicine, Uniformed Services University of the Health Sciences, Bethesda, MD 20814, USA; 5 Armed Forces Radiobiology Research Institute, Uniformed Services University of the Health Sciences, Bethesda, MD 20814, USA

**Keywords:** colony-forming units, gamma-tocotrienol, hematopoietic progenitors, nonhuman primates, radiation countermeasure, total-body irradiation

## Abstract

Radiation exposure causes acute damage to hematopoietic and immune cells. To date, there are no radioprotectors available to mitigate hematopoietic injury after radiation exposure. Gamma-tocotrienol (GT3) has demonstrated promising radioprotective efficacy in the mouse and nonhuman primate (NHP) models. We determined GT3-mediated hematopoietic recovery in total-body irradiated (TBI) NHPs. Sixteen rhesus macaques divided into two groups received either vehicle or GT3, 24 h prior to TBI. Four animals in each treatment group were exposed to either 4 or 5.8 Gy TBI. Flow cytometry was used to immunophenotype the bone marrow (BM) lymphoid cell populations, while clonogenic ability of hematopoietic stem cells (HSCs) was assessed by colony forming unit (CFU) assays on day 8 prior to irradiation and days 2, 7, 14, and 30 post-irradiation. Both radiation doses showed significant changes in the frequencies of B and T-cell subsets, including the self-renewable capacity of HSCs. Importantly, GT3 accelerated the recovery in CD34^+^ cells, increased HSC function as shown by improved recovery of CFU-granulocyte macrophages (CFU-GM) and burst-forming units erythroid (B-FUE), and aided the recovery of circulating neutrophils and platelets. These data elucidate the role of GT3 in hematopoietic recovery, which should be explored as a potential medical countermeasure to mitigate radiation-induced injury to the hematopoietic system.

## 1. Introduction

Exposure to hazardous radiation, intentional or accidental, can be deleterious, and depending on the absorbed dose may result in sickness, morbidity, and even death [1,2]. Acute radiation syndrome (ARS) occurs in humans following total- or significant partial-body exposure to ionizing radiation and is very much dose-dependent as seen in various sub-syndromes, including the hematopoietic sub-syndrome (H-ARS, 2–6 Gy), gastrointestinal sub-syndrome (GI-ARS, 6–8 Gy) and the neurovascular sub-syndrome (>8 Gy) [3]. Both hematopoietic and GI tissues are the most susceptible to radiation toxicity [4,5,6,7]. The H-ARS mainly arises due to the profound damage to actively proliferating stem cells (highly radiosensitive) in the bone marrow (BM), resulting in pancytopenia, increased risk of secondary infections and increased vascular permeability and hemorrhage in vital organs [8,9,10,11,12]. In addition, hematopoietic damage can lead to altered/abnormal function of the immune system, one of the adverse effects of radiation exposures that can last long-term [13,14]. Therefore, recovery and survival following high doses of radiation is mainly dependent on HSC reserves and its self-renewal ability to repopulate hematopoietic progenitor cells (HPCs), and thereby, restore functional immune cell repertoires, facilitating animal survival [7,15,16].

Efforts have been ongoing over the past several decades to discover agents that can enhance survival after radiation exposure that are safe, non-toxic, and effective, and can be easily distributed and administered. However, to combat H-ARS, only four agents have been approved by the Food and Drug administration (FDA), and all are radiomitigators [17,18,19,20]. To date, there is no approved radioprotector that can be used prior to radiation exposure for protection from the deleterious effects of radiation exposure. In recent years, several natural products have been evaluated and compared to their synthetic analogue for the prevention and treatment of radiation injuries [21]. Amongst them, vitamin E is a well-known antioxidant that can regulate peroxidation reactions and scavenge free-radicals, one of the major mediators of radiation toxicity [22,23]. This family comprises of eight different isoforms, including the α, β, γ, and δ tocopherols and corresponding tocotrienols, collectively known as tocols. Several studies have shown that tocotrienols are superior antioxidants compared to tocopherols [21,24,25,26].

Amongst the tocols, GT3 has been shown to be one of the most promising radioprotectors tested to date. It is a potent antioxidant, free radical scavenger [23,27], and is an inhibitor of 3-hydroxy-3-methylglutaryl-coenzyme A reductase, a pathway in cholesterol synthesis [28,29]. Several studies have demonstrated the radioprotective efficacy of GT3 in mice and NHPs when administered 24 hours (h) before TBI [27,30]. GT3 has also been shown to accelerate hematopoietic recovery in murine as well as NHP models [30,31,32]. GT3 treatment enhanced HPCs in the BM of irradiated mice [32]. In addition, GT3 induced hematopoietic growth factors and several other cytokines, particularly G-CSF, which is critical in stem cell mobilization and hematopoietic recovery [33,34,35], in addition to reducing DNA damage of myeloid/lymphoid hematopoietic progenitors [32]. The administration of G-CSF antibody abrogated its radioprotective efficacy [34]. In a recent study in an H-ARS model, both GT3 and α-tocopherol succinate (another tocol), were found to reverse serum and jejunum protein expression changes [36,37]. Furthermore, we recently reported radioprotective function of GT3 in intestinal epithelial and crypt cells in an NHP model exposed to a supralethal dose of radiation [38,39]. GT3 also modulated irradiation-induced transcriptomic changes [40], and showed promising hematopoietic recovery in the irradiated NHPs [31]. Importantly, GT3 is currently under advanced development as a radioprotective MCM for use as pre-exposure prophylaxis for H-ARS [27].

In this study, we evaluated the radioprotective efficacy of GT3 against radiation-induced hematopoietic injury and its implication on H-ARS in an NHP model exposed to total-body radiation of 4 and 5.8 Gy. While radiation exposure can be lifesaving in certain clinical settings, it can also potentially result in adverse effects to the hematopoietic and immune cells. Several studies have evaluated hematopoietic and immune cells in various radiation models including NHPs; most of these have focused on immune cell populations in the peripheral blood (B and/or T cells), with few studies characterizing these populations in hematopoietic organs [41,42,43]. However, to our knowledge, this is the first report with GT3 demonstrating modulation of major immune cell populations (CD34^+^ hematopoietic stem cell (HSCs), T cells, regulatory T (Treg) cells, B cells, natural killer (NK) cells, monocytes, and granulocytes) in the bone marrow of irradiated NHPs, in addition to evaluating GT3-induced peripheral blood recovery. Results of this study demonstrate that both 4 and 5.8 Gy radiation doses significantly reduced the percentage of hematopoietic stem and progenitor cells (HSPCs) and other immune cells including the self-renewable capacity of HSCs. GT3 treatment accelerated the recovery in CD34^+^ cells and increased HPCs function as shown by improved recovery of colony forming unit-granulocyte macrophages (CFU-GM) and burst-forming units erythroid (B-FUE). GT3 treatment also aided in the recovery of neutrophils and platelets. Taken together, these data suggest that GT3 enhances hematopoietic recovery by stimulating/preserving the self-renewable capacity of HSCs. GT3 should be explored further as a potential radioprotector to mitigate radiation-induced injury to the hematopoietic system.

## 2. Results

### 2.1. Effects of GT3 on Complete Blood Count (CBC) in NHPs Exposed to 4 and 5.8 Gy TBI

**Figure 1:**
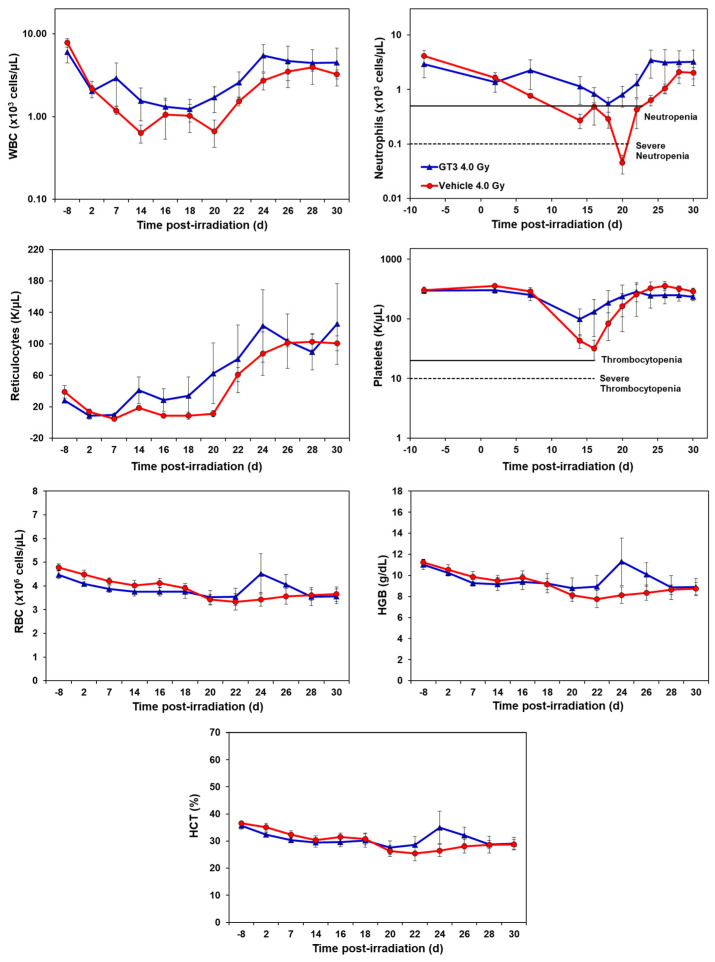
Effects of GT3 on complete blood count (CBC) in NHPs exposed to 4 Gy TBI. NHPs received GT3 (37.5 mg/kg) or vehicle 24 h prior to irradiation. Peripheral blood was collected at various time points. Cells were counted utilizing a Bayer Advia-120 cell counter. Data are expressed as mean ± SEM.

**Figure 2:**
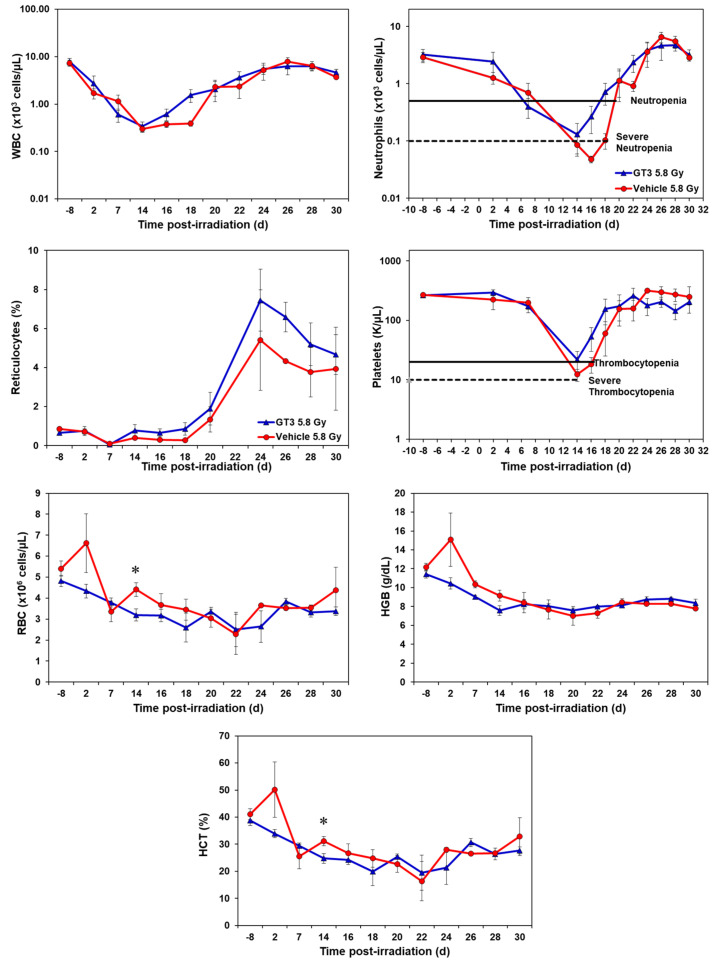
Effects of GT3 on complete blood count (CBC) in NHPs exposed to 5.8 Gy TBI. NHPs received GT3 (37.5 mg/kg) or vehicle 24 h prior to irradiation. Peripheral blood was collected at various time points. Cells were counted utilizing a Bayer Advia-120 cell counter. Data are expressed as mean ± SEM. * The difference between GT3- and vehicle-treated groups was significant when equal variance between groups was assumed (* *p* < 0.05).

GT3-treated animals showed an accelerated hematopoietic recovery in comparison to their respective vehicle group as shown in Figure 1 and Figure 2 and Supplementary Figures S1 and S2.

#### 2.1.1. Effects of GT3 on CBC Post 4 Gy Radiation

Compared to the vehicle, GT3-treated animals exhibited a higher level of WBC counts beginning at day 7 post-irradiation, which persisted until day 30. However, the average levels of both groups recovered close to baseline levels by day 30 (latest time point examined in this study). GT3-treated NHPs had a higher number of neutrophils, while the vehicle-treated group showed severe neutropenia. Interestingly, GT3 had improved reticulocyte recovery early on by day 14 in comparison to the vehicle-treated group; however, the difference between the treatments was not significant. Likewise, the platelet counts for the vehicle-treated group showed a more pronounced decrease in response to irradiation when compared to the GT3-treated group, but there was no significant difference between the treatment groups at any time point. In addition, there was no significant difference noted for RBC, hemoglobin (Hgb) and hematocrit (HCT) levels between the treatments. However, an increase in all three parameters peaked in the GT3-treated group on day 24 post-irradiation, but returned to levels similar to the vehicle-treated group by the end of the study. Monocyte and lymphocytes followed similar trends between groups throughout the study. The GT3-treated NHPs had higher levels of basophils by day 14 and returned to pre-exposure levels by day 30, while eosinophils showed increased levels by day 20 and remained elevated in this group for the remainder of the study (*p* < 0.05) (Supplementary Figure S1).

#### 2.1.2. Effects of GT3 on CBC Post 5.8 Gy Radiation

GT3-treated NHPs exposed to 5.8 Gy had higher levels of white blood cells (WBC) from day 16 onwards and continued until day 25, by which the counts for both groups returned to baseline (Figure 2). Neutrophil and platelet recovery was enhanced in the GT3-treated group compared to the vehicle. Reticulocytes were higher in the GT3-treated group from day 14 to day 30 when compared to the vehicle-treated group; however, the increase was not significant at any time point. GT3 did not seem to modulate RBC, HGB and HCT parameters at 5.8 Gy radiation. Even though monocytes, lymphocytes, and eosinophils showed a similar response curve post-radiation, the GT3-treated group showed an improved level when compared to the vehicle-treated group from day 24 onwards to day 30 (Supplementary Figure S2). Basophil concentrations were variable throughout the study in both groups and did not seem to show a drug-related response.

### 2.2. Effects of GT3 on Hematopoietic Injury in BM of NHPs Exposed to 4 and 5.8 Gy TBI

The changes in immune cell phenotype and hematopoietic stem cells in BM of GT3-treated animals were compared to their respective vehicle group and to unirradiated animals on day 8 prior to irradiation in each group. These observations are summarized below (Figure 3, Figure 4, Figure 5, Figure 6, Figure 7, Figure 8, Figure 9, Figure 10 and Figure 11).

#### 2.2.1. Leukocytes

**Figure 3:**
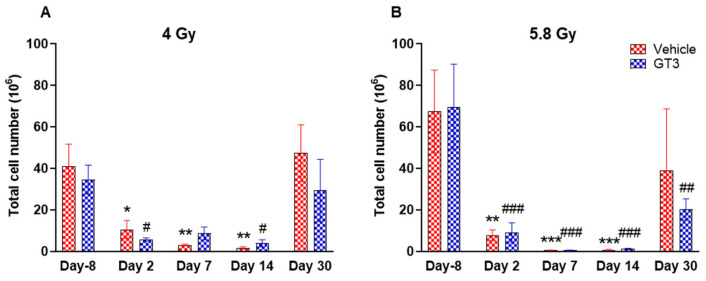
Effects of 4 Gy (**A**) and 5.8 Gy (**B**) on total leukocyte population in the BM of NHPs. NHPs received GT3 (37.5 mg/kg) or vehicle 24 h prior to irradiation (4 Gy and 5.8 Gy). BM was collected at various time points and expression of leukocytes were analyzed. Data are expressed as mean ± SEM. * *p* < 0.05, ** *p* < 0.01, *** *p* < 0.001 vs. vehicle day-8; ^#^ *p* < 0.05, ^##^ *p*< 0.01 ^###^ *p* < 0.001 vs. GT3 day -8.

A progressive decrease in the total number of WBC counts was observed in vehicle- and GT3-treated groups post-irradiation (Figure 3A,B). However, these numbers returned to normal by day 30 in both groups at 4 Gy (Figure 3A), and in the vehicle-treated group at 5.8 Gy (Figure 3B). These changes were statistically significant on days 2, 7 and 14 in vehicle-treated groups (74 to 96% decrease, *p* < 0.05 and *p* < 0.01), and on days 2 and 14 in GT3-treated groups (84 to 88% decrease, *p* < 0.05) when compared with day -8 at 4 Gy. These changes were more prominent at 5.8 Gy at all time points when compared to day -8 in the vehicle-treated groups except on day 30 (88 to 99% decrease day -8, *p* < 0.01 and *p* < 0.001) and in GT3-treated groups (71 to 99% decrease, *p* < 0.001 and *p* < 0.01) (Figure 3B). However, no statistical difference was observed between vehicle- and GT3-treated groups across the time points (Figure 3A,B).

#### 2.2.2. Hematopoietic Cells (CD45^+^)

**Figure 4:**
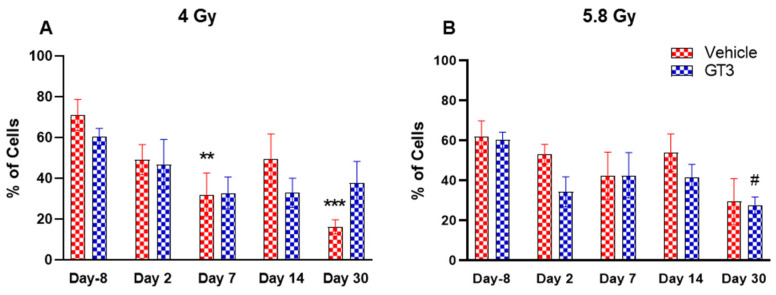
Effects of 4 Gy (**A**) and 5.8 Gy (**B**) on CD45 expression in BM of NHPs. NHPs received GT3 (37.5 mg/kg) or vehicle 24 h prior to irradiation (4 and 5.8 Gy). BM was collected at various time points and expression of CD45^+^ cells were analyzed. Data are expressed as mean ± SEM. ** *p* < 0.01, *** *p* < 0.001 vs. vehicle day-8; ^#^ *p* < 0.05 vs. GT3 day-8.

BM cells were labeled with CD45, a transmembrane protein tyrosine phosphatase located on most hematopoietic cells [44,45]. As shown in Figure 4A,B, radiation doses of 4 and 5.8 Gy TBI induced a decrease in hematopoietic cells in both vehicle- and GT3-treated groups. These changes were noted to be significant at day 7 and 30 in the vehicle-treated group at 4 Gy (compared to day-8, *p* < 0.01, *p* < 0.001, respectively) (Figure 4A). However, no significant differences were observed between vehicle- and GT3-treated groups across time points in both irradiated groups.

#### 2.2.3. Hematopoietic Stem Cells (CD34^+^CD45^+^)

**Figure 5:**
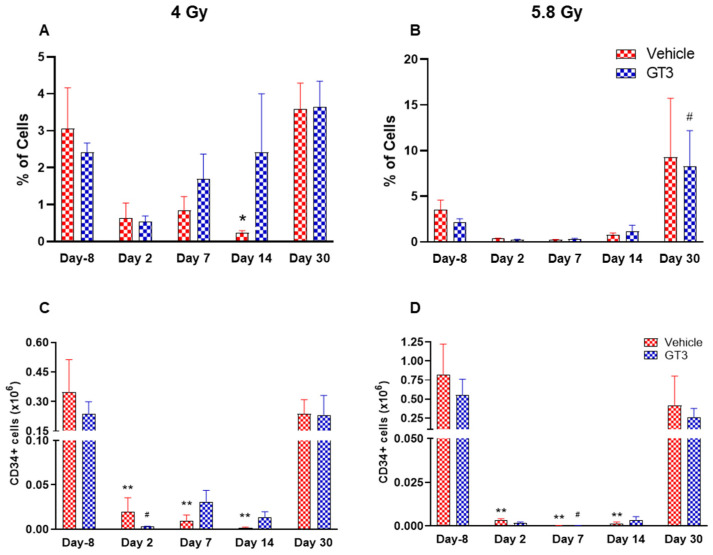
Effects of 4 and 5.8 Gy on CD34 percentages (**A**,**B**) and total cell counts (**C**,**D**) in BM of NHPs. NHPs received GT3 (37.5 mg/kg) or vehicle 24 h prior to irradiation (4 and 5.8 Gy). BM was collected at various time points and expression of CD34^+^ cells (**A**,**B**) and total number (**C**,**D**) were analyzed. Data are expressed as mean ± SEM. * *p* < 0.05, ** *p* < 0.01 vs. vehicle day-8; ^#^ *p* < 0.05 vs. GT3 day-8.

HSCs give rise to myeloid and lymphoid lineages and are responsible for maintaining the immune system [46,47]. CD34 is a marker of hematopoietic stem cells (HSCs) in humans and NHPs [41,44,45]. Radiation doses of 4 and 5.8 Gy TBI induced a dose-dependent reduction in percent positive and total number of HSCs on days 2, 7 and 14 in vehicle- and GT3-treated groups (Figure 5). Nevertheless, statistically significant differences were observed in percentages on day 14 (Figure 5A), and in the total number on days 2, 7 and 14 at 4 and 5.8 Gy (*p* < 0.01) in the vehicle group (Figure 5C,D). In contrast, in GT3-treated groups, the total number of these cells were significantly decreased only on day 2 at 4 Gy and on day 7 at 5.8 Gy (*p* < 0.01). Interestingly, GT3-treated groups showed an increase in CD34^+^ cells on days 7 and 14 at 4 Gy TBI, though the level of significance was not achieved (Figure 5A). Further, the percentage of HSCs were appreciably higher on day 30 in both vehicle- and GT3-treated groups in both doses of TBI (Figure 5A,B). However, at 5.8 Gy, there were only two animals in the vehicle-treated groups at day 30.

#### 2.2.4. B Cells (CD3^−^CD20^+^)

**Figure 6:**
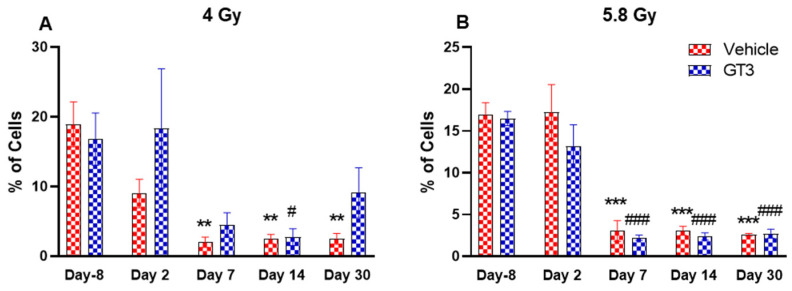
Effects of 4 Gy (**A**) and 5.8 Gy (**B**) on B cell expression in BM of NHPs. NHPs received GT3 (37.5 mg/kg) or vehicle 24 h prior to irradiation (4 and 5.8 Gy). BM was collected at various time points and expression of B^+^ cells were analyzed. Data are expressed as mean ± SEM. ** *p* < 0.01, *** *p* < 0.001 vs. vehicle day-8; ^#^ *p* < 0.05, ^###^ *p* < 0.001 vs. GT3 day-8.

B cells are an essential component of the humoral immune response and are responsible for generating specific antibodies to protect against antigens at the site of infection. B-cells are generated in the bone marrow, migrate to the spleen to mature, and circulate through the body to be close to the infection site [48]. In NHPs, B cells are classified as CD3^-^CD20^+^ [41]. A distinct reduction in the frequency of B cells were noted on day 7, 14 and 30 in vehicle-treated groups at 4 Gy (87 to 89% decrease compared to day-8, *p* < 0.01) (Figure 6A), and in vehicle- and GT3-treated groups at 5.8 Gy post-TBI (81 to 86% decrease compared to day-8, *p* < 0.001) (Figure 6B). In contrast, in GT3-treated groups at 4 Gy, these changes were minor with no statistical significance except on day 14 (*p* < 0.05). This may be indicative of GT3-mediated protection from radiation-induced damage to BM cells. However, there was no significant difference noted between vehicle- and GT3-treated groups at any time points examined in both irradiated groups.

#### 2.2.5. T Cells (CD3^+^) and Subsets

As part of adaptive immunity, T cells play an important role in controlling and executing immune responses. These cells originate in the bone marrow, mature in the thymus, and circulate throughout the body. Upon antigen encounter, T cells further differentiate and mature in circulation [49]. Each subset of T cells has a distinct function and helps the immune response to achieve best efficacy.

**Figure 7:**
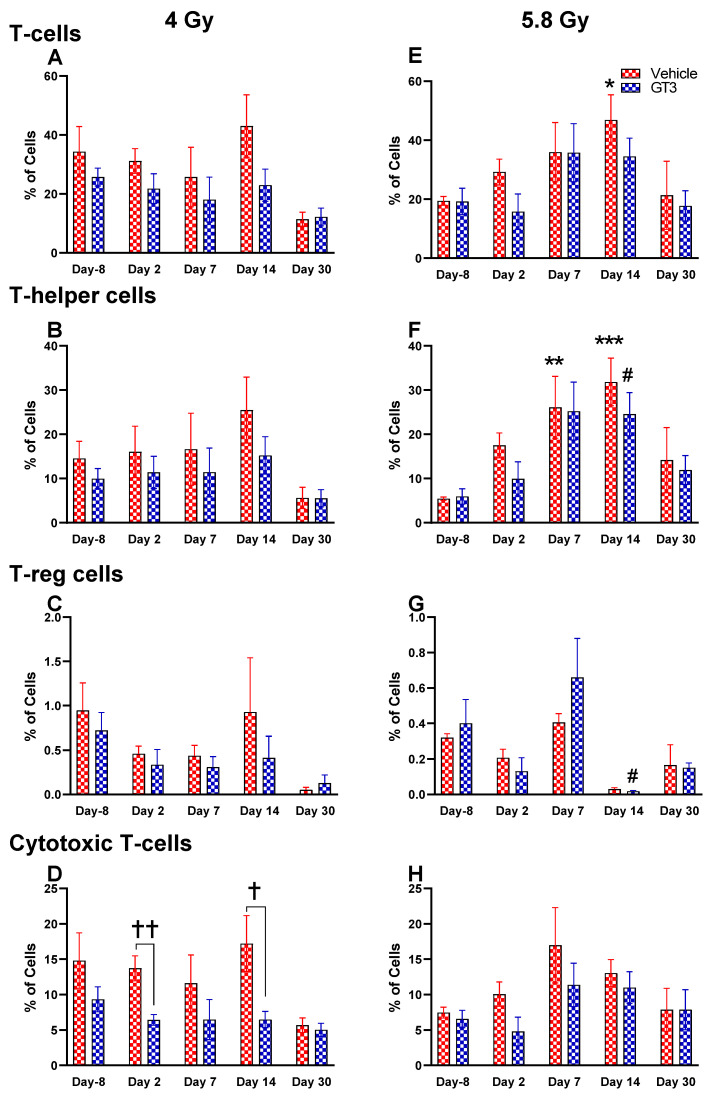
Effects of 4 and 5.8 Gy on T-cell (**A**,**E**), T-helper cell (**B**,**F**), T-reg cell (**C**,**G**), and cytotoxic T-cell (**D**,**H**) expression in BM of NHPs. NHPs received GT3 (37.5 mg/kg) or vehicle 24 h prior to irradiation (4 and 5.8 Gy). BM was collected at various time points, and the expression of T^+^ cells and their subsets were analyzed. Data are expressed as mean ± SEM. * *p* < 0.05, ** *p* < 0.01, *** *p* < 0.001 vs. vehicle day-8; ^#^ *p* < 0.05 vs. GT3 day-8; ^†^ *p* < 0.05, ^††^ *p* < 0.01, vehicle vs. GT3 at Day 2 and 14.

**Figure 8:**
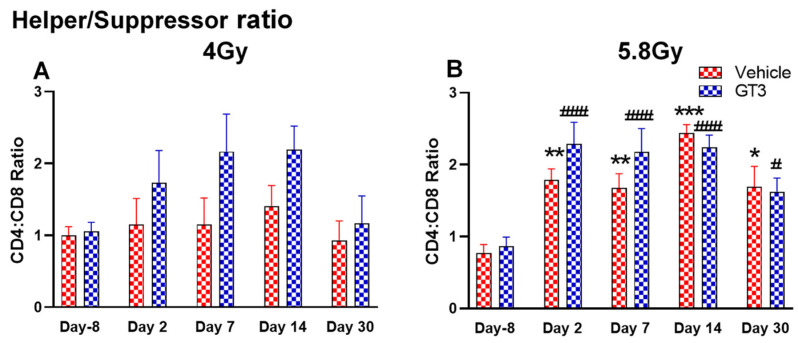
Effects of 4 Gy (**A**) and 5.8 Gy (**B**) on CD4:CD8 ratio in BM of NHPs. NHPs received GT3 (37.5 mg/kg) or vehicle 24 h prior to irradiation (4 and 5.8 Gy). BM was collected at various time points and the expression of the CD4:CD8 ratio was analyzed. Data are expressed as mean ± SEM. * *p* < 0.05, ** *p* < 0.01, *** *p* < 0.001 vs. vehicle day-8; ^#^ *p*< 0.05, ^###^ *p*< 0.001 vs. GT3 day-8.

Functionally diverse T cells can be divided into helper T cells (T_H_), cytotoxic T cells (T_C_), regulatory T cells (Treg), and unconventional subtypes, such as NKT cells and γ/δ T cells. Typically, T cells are identified by their expression of CD3 together with either CD4 (T_H_) or CD8 (T_C_) [41,50]. Additional markers such as CD25 (Treg) and CD56 (natural killer T cells) were used to further distinguish subtypes [41]. Here, we have immunophenotyped some of the major subsets which are discussed later in the study. At 4 Gy post-TBI, no significant changes were noted in the frequencies of T cells, T_H_, Treg and T_C_ subsets in vehicle- and GT3-treated groups when compared to day-8 (Figure 7A–D). However, the percentage of these cell subtypes appeared lower in GT3-treated groups compared to the vehicle. In addition, a significant decrease was observed in the T_C_ population at days 2 and 14 between vehicle- and GT3-treated groups (*p* < 0.01, *p* < 0.05, respectively) (Figure 7D). However, no significant change was observed in T_C_ cells in vehicle- and GT3-treated groups at 5.8 Gy TBI (Figure 7H). At 5.8 Gy post-TBI, a general increase in the frequencies of T cells was noted on days 2, 7 and 14 in both vehicle- and GT3-treated groups, with the vehicle being significant on day 14 (*p* < 0.05) (Figure 7E). Likewise, a significant increase was also seen in T_H_ cells on days 7 and 14 in the vehicle-treated group, and on day 14 in GT3-treated groups (*p* < 0.01, *p* < 0.001 and *p* < 0.05, respectively) (Figure 7F). While in Tregs, the suppressors of immune response, the only significant change observed was at day 14 in GT3-treated animals (*p* < 0.05) (Figure 7G). No significant change was observed in T_C_ cells in vehicle- and GT3-treated groups at 5.8 Gy (Figure 7H). Furthermore, we assessed the CD4:CD8 ratio, which indicates the overall immune health of an individual, and compared the values with the ratio at day -8 in both groups (4 and 5.8 Gy). A highly significant increase was seen in the ratio of these cells at all-time points tested in both vehicle- and GT3-treated groups at 5.8 Gy TBI (in vehicle-132%, 117%, 216%, 119% on days 2, 7, 14 and 30; in GT3–162%, 150%, 157%, 87% on days 2, 7, 14 and 30, respectively) (Figure 8B). An increase in the CD4:CD8 ratio was also observed in GT3-treated groups in 4 Gy TBI at days 2, 7 and 14 when compared to the vehicle; however, statistical significance was not achieved (Figure 8A).

#### 2.2.6. NK Cells and Subsets

**Figure 9:**
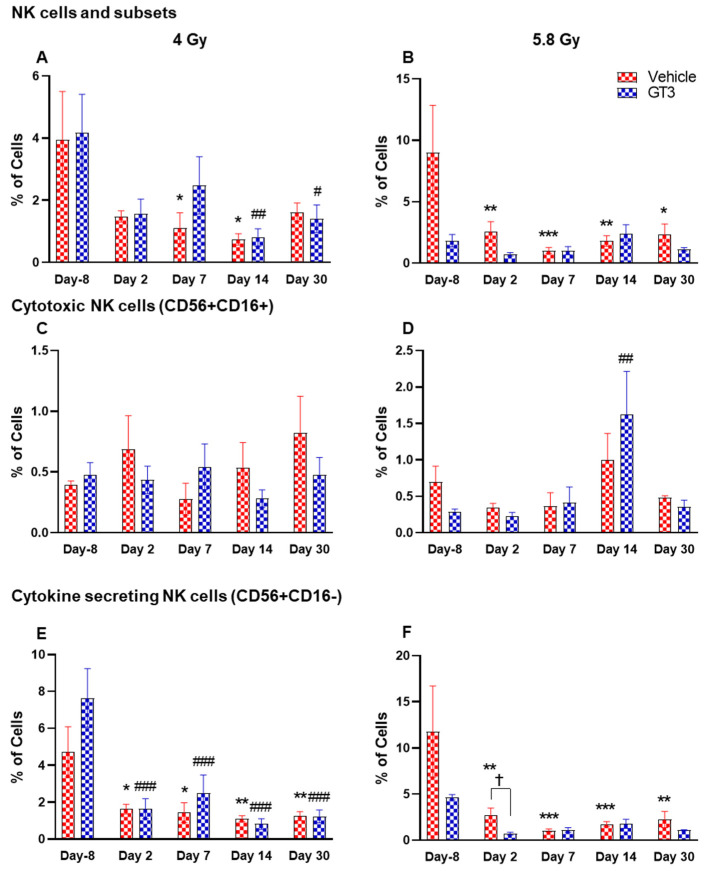
Effects of 4 and 5.8 Gy on NK cell (**A**,**B**), cytotoxic NK cell (**C**,**D**), and cytokine secreting NK cell (**E**,**F**) expression in BM of NHPs. NHPs received GT3 (37.5 mg/kg) or vehicle 24 h prior to irradiation (4 and 5.8 Gy). BM was collected at various time points and the expression of NK cells and their subsets were analyzed. Data are expressed as mean ± SEM. * *p* < 0.05, ** *p* < 0.01, *** *p* < 0.001 vs. vehicle day-8; ^#^ *p* < 0.05 ^##^ *p* < 0.01 ^###^ *p* < 0.001 vs. GT3 day -8. *p* < 0.05, Vehicle vs. GT3 at day 2.

Natural killer (NK) cells are important cells of the innate immune system that are involved in the early defense against foreign and virally transformed cells. Neural cell adhesion marker (N-CAM), CD56, is a marker to define NK cells in humans and NHPs, which consist of 2–12% of the lymphocyte population [41,51,52]. In this study, CD3^−^CD56^+^ cells were considered as NK cells. Time- and dose-dependent changes were observed in the percentage of NK cells, which were greatly reduced in vehicle-treated animals post-irradiation. These changes were statistically significant on days 7 and 14 at 4 Gy (*p* < 0.05), and at all time points at 5.8 Gy when compared with day-8 (*p* < 0.01, *p* < 0.001, *p* < 0.01, *p* < 0.05, respectively) (Figure 9A,B). However, in the GT3-treated group, a significant decrease was observed only on days 14 and 30 at 4 Gy (Figure 9A) (*p* < 0.01 and *p* < 0.05, respectively), and no significant difference was observed in 5.8 Gy treated animals compared to day-8 (Figure 9B). The delayed radiation-induced effect on the percent of NK cells at 4 Gy and no significant difference at 5.8 Gy may be linked to GT3-mediated protection.

Further, CD16 (FcγRIII) expression on CD56^+^ cells defines two populations. In healthy animals, the CD16^+^ NK cells are primarily cytotoxic while CD16^-^ are predominantly cytokine-secreting, which regulate other cells in the immune system. In CD56^+^CD16^+^ (cytotoxic cells), no significant difference was observed in this subset of NK cells at 4 Gy (Figure 9C), except a significant increase was seen on day 14 in GT3-treated animals at 5.8 Gy (*p* < 0.01) (Figure 9D). However, a remarkable reduction in the percentage of CD56^+^CD16^-^ expressing cells was noticed at all time points in both vehicle- and GT3-treated groups at 4 Gy (Figure 9E), and in vehicle-treated groups at 5.8 Gy compared to day-8 (Figure 9F). Though a significant difference was not observed in GT3-treated animals at 5.8 Gy TBI when compared to day-8, a significant reduction was observed on day 2 in GT3-treated animals compared to the vehicle (*p* < 0.05) (Figure 9F).

#### 2.2.7. Granulocytes and Monocytes (CD11b^+^)

**Figure 10:**
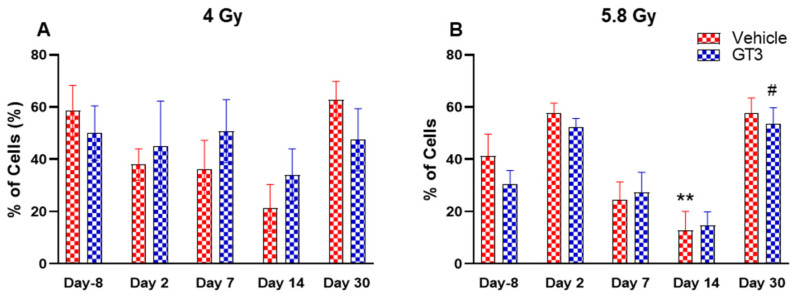
Effects of 4 Gy (**A**) and 5.8 Gy (**B**) on granulocyte and monocyte expression in BM of NHPs. NHPs received GT3 (37.5 mg/kg) or vehicle 24 h prior to irradiation (4 and 5.8 Gy). BM was collected at various time points and the expression of granulocytes and monocytes were analyzed. Data are expressed as mean ± SEM. ** *p* < 0.01 vs. vehicle day-8; ^#^ *p* < 0.05 vs. GT3 day-8.

Granular leukocytes or granulocytes include neutrophils, eosinophils and basophils, and are the components of the innate immune system. A large population of these cells is present in the BM of NHPs [41,45]. Based upon size and granularity, these cells can be differentiated on forward scatter and side scatter plots. In the present study, these cells and the monocytes, which are agranular myeloid cells, were differentiated by granularity, size and staining with CD11b. Although the percentage of granulocytes and monocytes appeared reduced in vehicle-treated animals on days 2, 7 and 14 at 4 Gy (Figure 10A) and on days 7 and 14 at 5.8 Gy TBI, it was significantly reduced only on day 14 at 5.8 Gy TBI (*p* < 0.01) (Figure 10B). In contrast, the percentages of these cells did not change much in GT3-treated animals at 4 Gy and 5.8 Gy TBI; however, a significant increase was noted on day 30 at 5.8 Gy (*p* < 0.05) (Figure 10B).

### 2.3. Effects of GT3 on Radiation-Induced Decrease in BM HSPCs Exposed to 4 and 5.8 Gy TBI

**Figure 11:**
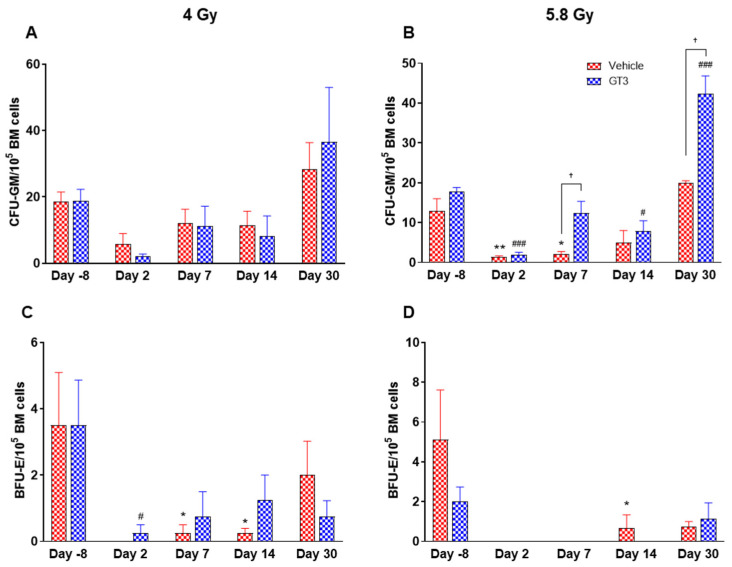
Effects of 4 and 5.8 Gy on CFU-GM (**A**,**B**) and BFU-E (**C**,**D**) counts post 14-day culture. NHPs received GT3 (37.5 mg/kg) or vehicle 24 h prior to irradiation (4 and 5.8 Gy). BM cells were collected at various time points and clonogenic function of HSCs were measured by CFU assay for colony forming unit-granulocyte macrophage (CFU-GM) (**A**,**B**), and burst-forming unit-erythroid (BFU-E) (**C**,**D**). Data are shown as the mean ± SEM. * *p* < 0.05, ** *p* < 0.01 vs. vehicle day-8; ^#^ *p* < 0.05, ^###^ *p* < 0.001vs. GT3 day-8. ^†^ *p* < 0.05, Vehicle vs. GT3.

Radiation-induced HSC damage influences all lineages of blood cells. Protecting and rescuing these stem cells are vital to mitigate radiation injury [7,53]. One of the important indicators for hematopoiesis and hematopoietic recovery are the numbers of CFUs. To evaluate the effects of GT3 on HSPCs function, CFU assays were employed to assess the colony forming abilities of HSPCs in NHPs exposed to 4 and 5.8 Gy TBI at days-8, 2, 7, 14 and 30. Both radiation doses decreased the numbers of CFU-GM and BFU-E compared to day-8, noted as early as day 2 and persisting until day 14 (Figure 11A–D). On the contrary, GT3-treated animals exposed to 4 Gy TBI showed an increase in CFU-GM (day 30), and BFU-E (day 7 and 14); however, these levels did not reach significance (Figure 11A,C). Interestingly, when compared with their respective vehicle-treated group, GT3 significantly increased CFU-GM from day 7 onwards in animals exposed to 5.8 Gy TBI, which persisted until day 30 (*p* < 0.05) (latest time point examined in this study), while BFU-E was drastically reduced post-radiation (Figure 11B,D). These data suggest that GT3 may ameliorate radiation-induced acute hematopoietic damage by enhancing hematopoiesis and facilitating stem cell regeneration.

## 3. Discussion

Exposure to total-body or partial-body radiation at doses greater than 1 Gy may result in ARS with significant hematopoietic damage [2,54]. The H-ARS is typically characterized by serious cytopenia and suppression of the hematopoietic compartments [10,55]. Even a sublethal dose of radiation can result in immunosuppression due to a reduced number of functional blood cells [6]. Importantly, delayed immune reconstitution/poor recovery from severe immunosuppression remains a major cause of morbidity associated with myelosuppression [56,57,58]. Hence, the protection or the reconstitution of the hematopoietic stem cell is a priority for effective thymopoiesis and for patient management [59,60,61]. The NHP has been a useful model for studies of ARS and is often used for evaluation of immune function and the development of radiation medical countermeasures (MCM) [57,62,63,64,65,66,67,68,69,70]. Most importantly, NHPs closely resemble humans and exhibit strong similarities with respect to pathophysiology and the immune system, and they also share genetic homology with more than 95% DNA sequence similarity [62,65,71,72]. Similar to humans, NHPs show an acute loss of circulating granulocytes and B cells, CD4^+^, and CD8^+^ T cells days following radiation with delays in the recovery of T cells [56,57]. This study investigated the potential of GT3 in accelerating hematopoietic recovery in NHPs exposed to 4 and 5.8 Gy TBI. The dynamic changes in the immune cell phenotype in the irradiated BM treated with or without GT3 were assessed over the course of 30 days. In addition, we also monitored peripheral blood cell recovery following irradiation.

GT3 has demonstrated radioprotective efficacy in mice as well as NHPs and was highly effective when administered 24 h before TBI [27,31,38,73]. Moreover, GT3, a potent antioxidant and a free radical scavenger [23,27], is currently under advanced development as a radioprotective MCM for use as pre-exposure prophylaxis for H-ARS [31]. This can be beneficial for first responders, military personnel, and individuals anticipating radiation exposure. Importantly, in an NHP model, a single administration of GT3 was found to be equally effective (with no supportive care) in improving hematopoietic recovery compared to multiple doses of Neupogen/Leukine and two doses of Neulasta (with complete supportive care) [25]. Notably, GT3 has successfully shown its radioprotective efficacy by enhancing hematopoietic recovery in NHP models and its ability to preserve the regenerating ability of the HSC [31]. Here, we report that a dose of 4 and 5.8 Gy TBI increased severity and duration of neutropenia and thrombocytopenia and reduced circulating reticulocytes. Further, radiation induced a dose-dependent significant reduction in BM leukocyte populations including decreased percentages of HSCs and impaired HSPCs function. These changes were noted as early as day 2 (the earliest time point examined in this study). GT3 treatment was associated with enhanced recovery of HSCs and HSPCs function as evident by increased CFUs with improved neutrophil, platelet and reticulocyte recovery in the peripheral blood.

Studies have extensively documented radiation-induced depletion of cells in the peripheral blood [57,74,75] and bone marrow [76], including the decrease in weight of lymphoid organs [77], which is dependent on the dose absorbed by the biological tissue. HSCs are vital for producing and maintaining blood cells in circulation. Studies have shown lymphocytes to be highly sensitive to radiation and diminish first from circulation followed by neutrophils and platelets following exposure to radiation (>2 Gy). Severe loss of lymphocytes, neutrophils, and platelets resolved within 30 days in NHPs exposed to 6 Gy TBI [57], while myeloid recovery were markedly different. Interestingly, GT3-treated mice showed almost complete recovery of peripheral blood monocytes, neutrophils and platelets by day 16 post-TBI [30]. Likewise, GT3 treatment at 5.8 and 6.5 Gy TBI reduced the severity and duration of neutropenia and thrombocytopenia in an NHP model [31]. This is in concordance with our observation where GT3-treated NHPs showed improved WBC recovery in addition to exhibiting decreased severity and duration of neutropenia and thrombocytopenia; however, this improvement did not reach the level of significance. This may be due to the small sample size of four animals per group. A future study utilizing a larger sample size is clearly warranted.

The BM serves as a reservoir for two multipotent stem cell populations: the hematopoietic (HSC) and mesenchymal stem cells (MSC). HSCs control the immune cell lineages including the myeloid and lymphoid lineages, which play an important role for maintaining the immune system [46,78]. Myeloid cells comprise of neutrophils, basophils, eosinophils, macrophages, monocytes, erythrocytes and platelets, whereas the lymphoid cells include T-cells, B-cells and natural-killer cells. Radiation exposures of high to sub-lethal doses can significantly compromise these cells, thereby influencing the proper function of the BM population. Studies have been completed that describe the immune cell depletion and recovery consequent to high- dose partial-body irradiation with 5% BM sparing to low lethal dose TBI in NHP models [56,57,63,65]. Both the TBI and PBI model elucidated variable B- and T-cell recovery kinetics in peripheral blood, suggesting the dependence on the recovery of marrow-derived stem and progenitor cells, peripheral homeostatic expansion and thymopoiesis. These coordinated requirements predict a prolonged recovery time following high to sub-lethal doses of radiation [57,65]. However, a majority of these studies have focused on evaluating immune cells in peripheral blood, with a few studies focusing on lymphoid organs such as spleen/thymus/BM [44,77]. Recently, we have shown that NHPs exposed to radiation doses of 6.7 and 7.4 Gy TBI experienced significantly decreased total BM cells and CD45^+^ CD34^+^ hematopoietic stem/progenitors cells in the BM [44]. In the current study, radiation-induced damage was more prominent at 5.8 Gy versus 4 Gy in different immune cells present in the BM, including HSCs and progenitor cells as reflected in their percentages. Interestingly, studies have shown that irradiated cells traffic to the BM and bring about the reduction of HSCs and progenitor cells [79]. Notably, GT3 treatment enhanced HSCs recovery on days 7 and 14 at 4 Gy which persisted until day 30 (latest time point examined in this study), while 5.8 Gy showed an increase on day 30, thus suggesting GT3 induced a dose-dependent recovery. The recovery of HSCs is essential to ensure adequate production of B cell progenitors as well as HSC mobilization.

The number of CFUs, an important indicator for hematopoiesis/hematopoietic recovery, can assess radiation-induced impairment of hematopoietic compartment [10,55]. Moreover, available literatures support the notion that various colony stimulating factors such as G-CSF, BFU-E, GM-CSF (granulocyte macrophage-colony stimulating factor), etc. significantly enhance recovery from radiation-induced hematopoietic injury, and may facilitate recovery of individuals experiencing ARS [10]. Here, we report that GT3 treatment increased the numbers of myeloid progenitors in the BM like CFU-GM and BFU-E, thus suggesting the ability of HSPCs to differentiate into granulocytes, monocytes and erythrocytes, and, thereby, may prevent acute hematopoietic damage induced by radiation. Overall, these results are consistent with previous studies in mice and NHPs, which show that GT3 protects hematopoietic tissue by preserving and/or inducing HSCs [31,32] and thereby promoting BM hematopoiesis.

The lymphocyte subsets (including CD3^+^, CD4^+^ and CD8^+^ T cells) and the NK cells are crucial in maintaining immune function [80]. CD3^+^ T cells are the only T lymphocytes in peripheral blood that have antitumor activity. While CD4^+^ T cells assist in B-cell maturation and are closely involved in cellular immunity [81], CD8^+^ T lymphocytes have cytotoxic activity and play a vital role in anti-tumor immunity [82]. The ratio of CD4^+^/CD8^+^ cells acts as an indicator of immune function and reflects the immunological status in cancer patients [80]. Studies have shown variable B- and T-cell recovery kinetics post-TBI in an NHP model [57,65]. Likewise, radiation-induced damage was also observed in cancer patients with a decreased CD4^+^/CD8^+^ ratio, suggesting that the immune system was greatly reduced [80]. Our study also showed dynamic changes in T cells and various subsets of T cells, such as CD4 and CD8 cells in response to GT3. A distinct increase in the ratio of CD4^+^/CD8^+^ in GT3-treated groups post-irradiation is noticeable, which suggests an increase in CD4^+^ cells. This increase may be attributed to cytokine production, which is required in the regulation of other immune cells in the bone marrow microenvironment. In addition, GT3 inhibited the radiation-induced decrease in B cells in 4 Gy treated groups, however, the effect was not observed at 5.8 Gy. Furthermore, NK cells are cytotoxic effectors of the innate immune system that are important in destroying tumor cells and contribute to natural resistance against microbial infections [83]. In addition, they are highly sensitive to radiation therapy, as shown in their decreased activity and numbers post-treatment [84]. Here, we observed a significant reduction in NK cells and its subset, with a pronounced effect noted in 5.8 Gy groups treated with or without GT3. Moreover, we have not performed any mechanistic studies to assess GT3 influence on their functionality, which is clearly warranted. A study by Zarcone et al., reported that following a radiation dose of 3 Gy, NK cells were able to form conjugates with the targets, but failed to undergo further activation [85].

It is important to note that two female animals in the 5.8 Gy irradiated group were euthanized on days 21 and 22 post-irradiation (met moribundity criteria defined in approved IACUC protocol) and both animals were vehicle-treated. No GT3-treated animals in the 5.8 Gy group met euthanasia criteria, suggesting beneficial effects of this candidate MCM for radiation injury. Earlier, we have demonstrated the radioprotective efficacy of GT3 against 5.8 and 6.5 Gy TBI in respect of hematopoietic recovery in NHPs [27,31]. A recent report reviewing various NHP (*Macacca mulatta*) studies where animals were irradiated with different doses of total-body radiation suggests that female NHPs have higher mortality than males at identical radiation doses [86]. Though our study is with a small number of animals, our observation is in agreement with this report.

In summary, GT3 promoted the recovery of HSCs and enhanced the functionality of BM hematopoietic progenitors, in addition to improving neutrophil and platelet recovery. Our data clearly shows that GT3 has a radioprotective function in the hematopoietic compartment in the context of H-ARS. Future studies are clearly warranted with an increased number of animals to explore in depth the potential of GT3 as a promising MCM to mitigate radiation-induced hematopoietic injury. Similar studies with GT3 using partial-body NHP exposure with a linear accelerator (LINAC) as a radiation source are in progress.

## 4. Materials and Methods

### 4.1. Animals

A total of 16 naïve rhesus macaques (*Macaca mulatta*, Chinese sub-strain, 8 males and 8 females) were used for this study. The animals were between 2.9–4.8 years of age, weighing 4–9 kg. A total of four NHPs were procured from the National Institutes of Health Animal Center (NIHAC, Poolesville, MD, USA). The remaining 12 NHPs were supplied by an NHP vendor (PrimGen, Hines, IL, USA). All animals were maintained and the study was performed in a facility accredited by the Association for Assessment and Accreditation of Laboratory Animal Care (AAALAC)-International. Animals were quarantined for six weeks prior to the initiation of the experiment. Animal housing, health monitoring, care, and enrichment during the experimental period have been described in detail earlier [31]. All procedures involving animals were approved by the Institutional Animal Care and Use Committee (BIOQUAL Inc., protocol #18-060) and the Department of Defense Animal Care and Use Review Office (ACURO). This study was accomplished in strict accordance with the recommendations made in the Guide for the Care and Use of Laboratory Animals [87].

### 4.2. Experimental Design

The objective of this study was to investigate hematopoietic injury as a result of total-body cobalt-60 gamma-irradiation and recovery by the use of a promising prophylactic radiation MCM, GT3, using an NHP model. Various assays conducted in this study are important to understand radiation-induced hematopoietic injury and its recovery. The sixteen NHPs of this study were divided into two groups; eight were administered GT3 (37.5 mg/kg) and the other eight were administered vehicle. Two different radiation doses, 4 Gy (sub-lethal) and 5.8 Gy (~LD30/60), for TBI were used. Four animals in each treatment group were exposed to 4 Gy and the other four with 5.8 Gy 24 h after drug/vehicle administration (Table 1).

### 4.3. Preparation and Administration of GT3 and Vehicle

GT3 and its vehicle were procured from Callion Pharma (Jonesborough, TN, USA). The GT3 was supplied as an injectable formulation at a concentration of 50 mg/mL and an olive oil formulation was used as the vehicle. Just prior to administration, the GT3 and vehicle formulations were thoroughly mixed using a laboratory vortex. GT3 or vehicle were administered at a dose of 37.5 mg/kg subcutaneously (*sc*) 24 h prior to total-body ^60^Co irradiation. Injections were accomplished with a sterile 21–25 gauge needle length of ¾–1” [38]. The site for injection (dorsal scapular region-midline) was prepared as a surgical site before the injection: hair was clipped using # 40 surgical clipper blade and the site was appropriately disinfected using either povidone iodine (betadine) or chlorhexidine and 70% alcohol. The drug or vehicle volume administered was based on the individual weight of the NHP.

### 4.4. Total-Body Irradiation

Food was withheld from each animal approximately 12–18 h before radiation exposure to minimize the occurrence of radiation-induced vomiting. To deliver the precise radiation dose, NHPs’ abdominal widths were measured with digital calipers. Approximately 30–45 min before irradiation, NHPs were administered 10–15 mg/kg of ketamine hydrochloride intramuscularly (*im*) for sedation, then placed in custom-made Plexiglas irradiation boxes and secured in a seated position. Two NHPs were placed on the irradiation platform facing away from each other and were then exposed to a radiation dose of either 4 or 5.8 Gy ^60^Co γ-radiation at a dose rate of 0.6 Gy/min from both sides of the core of the abdomen (bilateral, simultaneous exposure). The radiation field in the area of the NHP location was uniform within ±1.5%. Other details of irradiation and dosimetry are described earlier [88].

### 4.5. Peripheral Blood Collection and Complete Blood Counts

Blood was collected from a peripheral vessel (either saphenous or cephalic vein) as described earlier. The desired volume of blood was collected with a 3 mL disposable luer-lock syringe with a 25-gauge needle. Whole blood was collected in EDTA (ethylenediaminetetraacetic acid) blood collection tubes (Sarstedt Inc., Newton, NC, USA) for complete blood counts. Cells were counted utilizing a Bayer Advia 120 cell counter (Siemens, Malvern, PA, USA). Parameters evaluated include white blood cells, red blood cells, hemoglobin, hematocrit, platelet, neutrophil, and reticulocyte counts.

### 4.6. BM Collection

On the days of BM collection (8 days pre-irradiation and days 2, 7, 14 and 30 post-irradiation, day of irradiation considered as day 0), the NHPs were sedated with Telazol (5–10 mg/kg im) with a 22–25 G 5/8 -1” needle. The animals were fasted for 8–12 h before sedation. The site for BM aspiration was the iliac crest and the site was alternated between collection days (left and right hips). The hair surrounding the area was shaved and the site was cleaned using a surgical preparation solution. For collecting the BM, the Teleflex EZ-IO drill (Teleflex Inc. Morrisville, NC, USA) was used with a 25 mm needle attached to the driver. The iliac crest was palpated and the needle inserted at a 90° angle to the bone and advanced through the skin until contact was made with the bone. Then, using the driver, the bone was drilled. The trigger was immediately released when loss of resistance was felt. The stylet was then removed from the needle. Approximately 2 mL of BM was aspirated using a 20 mL syringe pre-filled with 8 mL of culture media (RPMI-1640 no phenol red, 5% fetal bovine serum (FBS) and 90 IU/mL of sodium heparin). The catheter was then removed by twisting clockwise and pulling straight out. Pressure was applied to control bleeding and a dressing applied as needed. One dose of buprenorphine (0.005–0.03 mg/kg, *im* or *sc*) was administered after the procedure for pain management. This procedure was carried out by trained personnel and under close supervision of a veterinarian. The BM sample was then transferred to a 50 mL sterile tube and additional media was added so that the total volume in the tube was approximately 45–50 mL. The samples were then shipped on wet ice via FEDEX priority overnight mail for analysis at the University of Arkansas Medical Sciences, Little Rock, AR. Two vehicle-treated animals in the 5.8 Gy group were euthanized (one on day 21 and the other on day 22 post-irradiation) based on IACUC protocol moribundity criteria. Thus, BM samples from these animals were not available for analysis on day 30 post-irradiation.

### 4.7. Flow Cytometry

BM cells were suspended in 10 mL lysing buffer (BD Biosciences, San Jose, CA, USA) and incubated for 15 min in the dark at room temperature. The cells were spun down at 200 g for 6 min, washed twice with 10 mL flow buffer (PBS with 2% FBS), and pellets were suspended in 1–2 mL flow buffer depending on the pellet size. Cell viability was assessed by the trypan blue dye exclusion method. For flow cytometry analysis, cells were incubated with Fc blocking reagent to block the nonspecific binding to Fcγ receptors on the cell surface (BD Biosciences) prior to staining. Finally, 1.5 × 10^6^ cells were labeled with a cocktail of various fluorochrome-conjugated antibodies for specific antigens (Supplementary Table S1), and incubated at 4 °C for 30 min. The fixable viability dye was included in the cocktail to exclude the dead cells during analysis. After incubation, the cells were washed with the washing buffer (PBS with 2% FBS and 0.1% NaN3) twice followed by fixation with 2% paraformaldehyde-PBS. Samples were acquired on a BD LSRFortessa cell analyzer operated by FACS Diva software (BD Biosciences). For each sample, approximately 1.0 × 10^6^ events were collected. Data were analyzed and illustrated using FCS express version 7.06 (De Novo software, Pasadena, CA, USA). Percent positive cells were calculated for each cell surface protein stained from the live cell gate of lymphocytes. The total number of specific cell types was calculated as per the formula (% positive cells × % of all cell gate × total BM count).

#### Gating Strategy

The compensation matrix and the gating strategy were established by running single stained controls, fluorescence minus one controls, and a cocktail of all immune cell markers on unirradiated NHP BM samples. With sequential gating, debris and cell aggregates were excluded by drawing an all cell gate, defined on a forward scatter and side scatter plot, and a lymph gate was created around the lymphocytes within the all cell gate. Using eFluor 506 live-dead stain, the dead cells were excluded by creating a live gate from the lymphocyte population, and only live cells were used for further analysis. Hematopoietic cells were defined as CD45^+^ and hematopoietic stem/progenitor cells as CD45^+^CD34^+^. Cell specific gates were created to identify different populations such as T cells (CD3^+^), cytotoxic T cells (CD8^+^), helper T cells (CD4^+^) and regulatory T cells (CD4^+^CD25^+^). B cells were defined as CD3^-^CD20^+^ and NK cells were considered as CD3^-^CD56^+^. Within the NK cells, different subsets were identified using a CD16 receptor expression pattern such as CD56^+^CD16^+^ and CD56^+^CD16^-^ populations. In addition, granulocytes and monocytes were defined as CD11b^+^ from granulocyte and monocyte gates within the all cell gate.

### 4.8. Bone Marrow CFU Assay

The CFU assays at days-8, 2, 7,14 and 30 were performed by culturing 1 × 10^5^ BM cells exposed to 4 and 5.8 Gy TBI using Methocult TM H4034 optimum (Stem Cell Technologies, Vancouver, BC, Canada). Colonies of burst-forming unit-erythroid (BFU-E) and CFU-granulocyte macrophage (CFU-GM) were scored on day 14 of the incubation according to the manufacturer’s protocol.

### 4.9. Statistical Analysis

Statistical analysis was performed using GraphPad Prism Version 9.1.0 (GraphPad Software, San Diego, CA, USA). Differences among treatment groups were assessed by two-way analysis of variance (ANOVA) tests to determine the interaction between vehicle and GT3 treatment at various time points. Dunnett multiple comparisons tests were performed to compare the mean at each time point from day-8 in each group separately. Pairwise comparisons were analyzed with the student’s *t*-test. *p* values of 0.05 or less were considered statistically significant.

## Figures and Tables

**Table 1:** Experimental design for 16 NHPs exposed to 4 and 5.8 Gy TBI.

Study Design with 16 NHPs					
Hematopoietic Study (Total-Body Irradiation, ^60^Co γ-Radiation, 0.6 Gy/min)					
NHP	Drug	Route	Dose	Frequency	Irradiation Dose (Gy)
4	GT3	*sc*	37.5 mg/kg	24 h prior to irradiation	4
4	Veh	*sc*	37.5 mg/kg	24 h prior to irradiation	4
4	GT3	*sc*	37.5 mg/kg	24 h prior to irradiation	5.8
4	Veh	*sc*	37.5 mg/kg	24 h prior to irradiation	5.8

## Data Availability

All relevant data, which supports the findings of the study, are within the manuscript and in the supplementary files.

## Supplementary Materials

The following supporting information can be downloaded at: https://www.mdpi.com/article/10.3390/ijms232416170/s1. Table S1: Antibodies used for the analysis of NHP-BM samples. Figure S1: Effects of GT3 on complete blood count (CBC) in NHPs exposed to 4 Gy TBI. NHPs received GT3 (37.5 mg/kg) or vehicle 24 h prior to irradiation. Blood was collected at various time points. Cells were counted using a Bayer Advia-120 cell counter. Data are expressed as mean ±SEM. * The difference between GT3-treated and vehicle-treated groups was significant when equal variance between groups was assumed (* *p* < 0.05). Figure S2: Effects of GT3 on complete blood count (CBC) in NHPs exposed to 5.8 Gy TBI. NHPs received GT3 (37.5 mg/kg) or vehicle 24 h prior to irradiation. Blood was collected at various time points. Cells were counted using a Bayer Advia-120 cell counter. Data are expressed as mean ±SEM. *The difference between GT3-treated and vehicle-treated groups was significant when equal variance between groups was assumed (* *p* < 0.05).
