# Supplementary material for: Gamma-Tocotrienol Modulates Total-Body Irradiation-Induced Hematopoietic Injury in a Nonhuman Primate Model
Source: Int J Mol Sci. 2022 Dec 18;23(24):16170. doi: 10.3390/ijms232416170 (PMC9784560; doi:10.3390/ijms232416170)
Supplement: Supplementary file 1 [file ijms-23-16170-s001.zip › ijms-2068381-supplementary.pdf]

Gamma-tocotrienol modulates total-body irradiation-induced hematopoietic injury in a nonhuman primate model

Tarun K. Garg [1,a], Sarita Garg [2, 3,a,b], Isabelle R. Miousse [3], Stephen Y. Wise [4,5], Alana D. Carpenter [4,5], Oluseyi O. Fatanmi [4,5], Frits van Rhee [1], Vijay K. Singh [4,5,*] and Martin Hauer-Jensen [2,*]

[1] UAMS Myeloma Center, University of Arkansas for Medical Sciences, Little Rock, AR;

[2] Division of Radiation Health, Department of Pharmaceutical Sciences, University of Arkansas for Medical Sciences, Little Rock, AR; [3] Department of Biochemistry and Molecular Biology, University of Arkansas for Medical Sciences, Little Rock, AR: [4] Division of Radioprotectants, Department of Pharmacology and Molecular Therapeutics, F. Edward Hébert School of Medicine, Uniformed Services University of the Health Sciences, Bethesda, MD; [5] Armed Forces Radiobiology Research Institute, Uniformed Services University of the Health Sciences, Bethesda, MD

[a] These authors contributed equally

[b] Present address: Department of Biochemistry and Molecular Biology, University of Arkansas for Medical Sciences, Little Rock, AR-72205

[*] Communicating author:
Martin Hauer-Jensen, MD, PhD
Division of Radiation Health
4301 West Markham, Slot 522-10
Little Rock, AR 72205
Phone: 501-686-7912
Fax: 501-421-0022
E-mail: mhjensen@uams.edu

Co-communicating author: Vijay K. Singh, vijay.singh@usuhs.edu, +1-301-295-2347

Supplementary Tables and Figures:

Supplementary Tables:

**Supplementary Table S1. Antibodies used for the analysis of NHP-BM samples**			
**Antibody**	**Fluorochrome**	**Clone**	**Manufacturer**
Live/dead	eFluor™ 506	N/A	eBioscience™
CD3	V450	SP34-2	BD Biosciences
CD4	PE-Alexa Fluor 700	S3.5	ThermoFisher Scientific
CD8	APC	LT8	ThermoFisher Scientific
CD25	Super Bright 702 (SB702)	CD25-4E3	eBioscience™
CD20	APC-H7	2H7	BD Biosciences
CD56	Super Bright 600 (SB600)	TULY56	eBioscience™
CD16	Super Bright 645 (SB645)	3G8	eBioscience™
CD34	PE	563	BD Biosciences
CD45	FITC	D058-1283	BD Biosciences
CD11b/Mac1	PE-Cy7	ICRF44	BD Biosciences

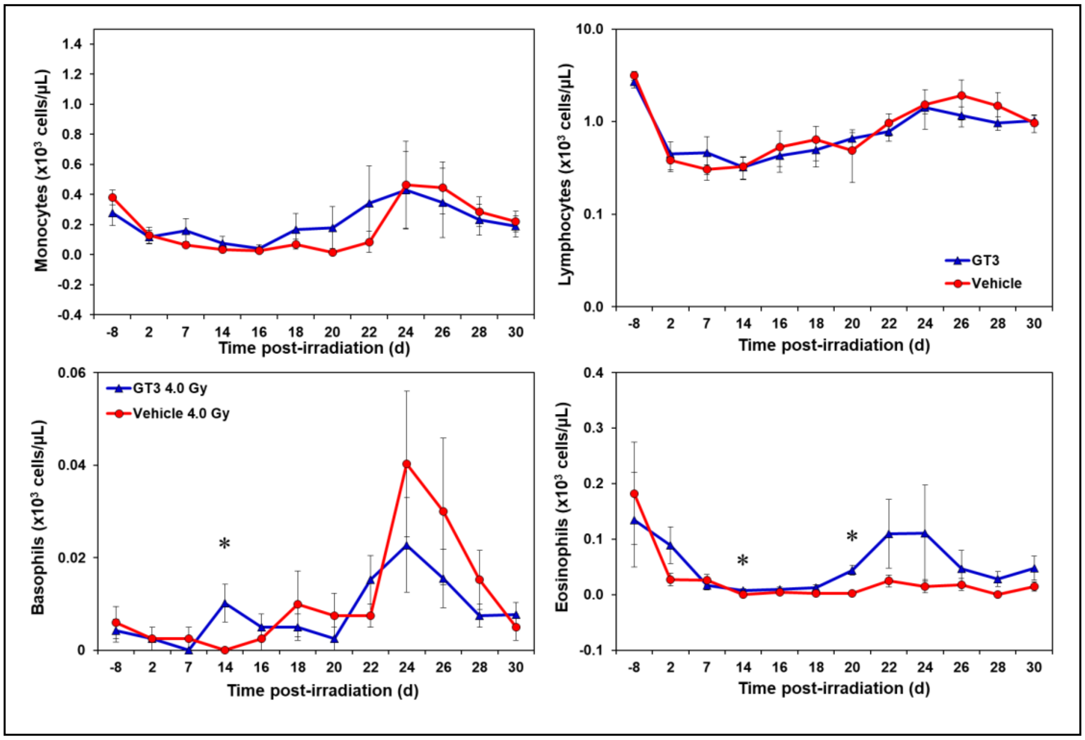

Supplementary Figure S1. Effects of GT3 on complete blood count (CBC) in NHPs exposed to 4 Gy TBI. NHPs received GT3 (37.5 mg/kg) or vehicle 24 h prior to irradiation. Blood was collected at various time points. Cells were counted using a Bayer Advia-120 cell counter. Data are expressed as mean ±SEM. *The difference between GT3-treated and vehicle-treated groups was significant when equal variance between groups was assumed (*$p < 0.05$).

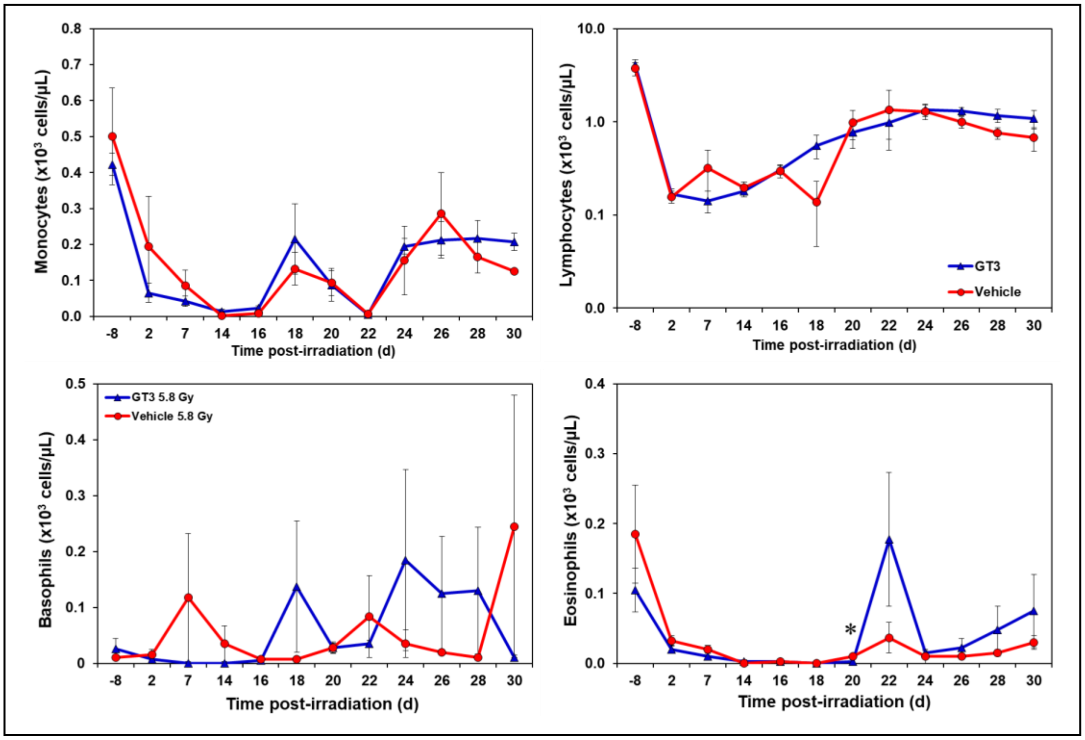

Supplementary Figure S2. Effects of GT3 on complete blood count (CBC) in NHPs exposed to 5.8 Gy TBI. NHPs received GT3 (37.5 mg/kg) or vehicle 24 h prior to irradiation. Blood was collected at various time points. Cells were counted using a Bayer Advia-120 cell counter. Data are expressed as mean ±SEM. *The difference between GT3-treated and vehicle-treated groups was significant when equal variance between groups was assumed (*$p < 0.05$).
